# Supplementary material for: Multiple Selection Criteria for Probiotic Strains with High Potential for Obesity Management
Source: Nutrients. 2021 Feb 24;13(3):713. doi: 10.3390/nu13030713 (PMC7995962; doi:10.3390/nu13030713)
Supplement: Supplementary file 1 [file nutrients-13-00713-s001.pdf]

Supplementary Material and Methods

Evaluation of the DPP-4-like peptidase activity of the strains tested

Strains were incubated in 96-well optical black plates (Nunc, ThermoFisher Scientific, Rochester, NY, USA) at 10^7 CFU/ml with 1 mM Gly-Pro-AMC (Merck, Molsheim, France), a specific substrate of the dipeptydil-peptidase-4 (DPP-4), in phosphate saline buffer pH 7.4 (PBS) in the presence or not of DPP-4 inhibitor(s). Ile-Pro-Ile (Diprotin A) was used at a final concentration of 0.5 mM and 5 μL of a commercial DPP-IV inhibitor coming from Millipore (Merck) was added in relevant wells. Fluorescence was recorded every 2 min for 20 min under constant stirring at 37 °C using a Xenius spectrofluorometer (Safas Monaco, Monaco). Excitation and emission wavelengths were set to 260 nm and 480 nm, respectively. Data were expressed as the slope from the linear regression of the percentage of fluorescence as a function of time (n=3).

Supplementary Tables

Table S1: *In vitro* abilities of the probiotic strains to restore or strengthen an epithelial barrier and to exhibit an anti-inflammatory profile (from Alard *et al*, 2017).

Strains designation	Improvement of Epithelial Barrier	IL-10 Induction	Anti-Inflammatory Profile (Il-10/IL-12)
B. lactis LA306	+	++	++
B. bifidum LA803	++	++++	++++
B. lactis LA804	+++	++++	++++
B. breve LA805	++	+++	++++
B. longum PI10	++	+++	++++
B. bifidum PI22	++	++++	++++
La. rhamnosus LA305	+	++	+++
Li. salivarius LA307	+	++	+++
L. gasseri LA806	++++	+/-	+/-
Li. salivarius PI2	+	++	++
Lp. plantarum PI3	+	+	++
L. acidophilus PI4	++	+	+/-
L. helveticus PI5	++++	+	++
La. casei PI8	+++	++	++
L. acidophilus PI11	++++	+/-	+/-
L. helveticus PI13	++	++	+
La. paracasei PI15	++	++	++
La. paracasei PI18	+++	++	++
Lp. plantarum PI19	+	+	+/-
La. casei PI20	++	+	+++
La. rhamnosus PI28	+	+/-	0
S. thermophilus PI21	0	+/-	0
Lc. lactis PI23	0	+/-	0

Table S2: Composition of the diets used in the study (g) (source: Research Diets).

	D12450B: 10% fat (LFD)	D12451: 45% fat (HFD)
Casein, 30 Mesh	200	200
L-Cystine	3	3
Corn Starch	315	72.8
Maltodextrin 10	35	100
Sucrose	350	172.8
Cellulose, BW200	50	50
Soybean Oil	25	25
Lard	20	177.5
Mineral MIX S10026	10	10
Dicalcium Phosphate	13	13
Calcium Carbonate	5.5	5.5
Potassium Citrate, 1 H_2O	16.5	16.5
Vitamin MIX V10001	10	10
Choline Bitartrate	2	2
FD&C Yellow Dye #5	0.05	0
FD&C Red Dye #40	0	0.05

Table S3: Forward and reverse primers′ sequences used in the study.

bact-F	5′- CTAAGGCCAACCGTGAAAAC -3′
bact-R	5′- ACCAGAGGCATACAGGGACA -3′
tnfa-F	5′-CCCTCACACTCAGATCATCTTCTC -3′
tnfa-R	5′-GGCTACAGGCTTGTCACTCG -3′
cd36-F	5′-TCATTGCAACTGAGTGG -3′
cd36-R	5′-TCATAAGCTCTGTGTCAG -3′
cd68-F	5′-TGGCGGTGGAATACAATGTG -3′
cd68-R	5′-GATGAATTCTGCGCCATGAA -3′
mcp1-F	5′-TCAGCCAGATGCAGTTAACGC -3′
mcp1-R	5′-TGATCCTCTTGTAGCTCTCCAGC -3′
hcrt-F	5′-CCGTAACTACCACCGCTTTAGC -3′
hcrt-R	5′-AAGAGACTGACAGCGGCGAG -3′
pomc-F	5′-CCGGTGAAGGTGTACCCCAA -3′
pomc-R	5′-TCCAAGCCTAATGGCCGCT -3′
lep-F	5′-AGGATGACACCAAAACCCTCAT -3′
lep-R	5′-AGTCCAAGCCAGTGACCCTCT -3′
lepR-F	5′-CAGTGTGCGCTGGTTCTGTC -3′
lepR-R	5′-TGTGAGGAGGTACGTGGTGAAG -3′
fabp1-F	5′-CCAATTGCAGAGCCAGGAGA -3′
fabp1-R	5′-CCCCTTGATGTCCTTCCCTTT -3′
tgr5-R	5′-CCCAACTTTTGTTTCCTTTCCC -3′
tgr5-F	5′-TAGCCGCACACTTCCATGG -3′
gln-R	5′-AGGGACCTTTACCAGTGATGT -3′
gln-F	5′-AATGGCGACTTCTTCTGGGAA -3′

Table S4. Definition of bile acid clusters and ratios.

Bile acid cluster	Acronym and definition
Free cholic acid	CA
Free chenodeoxycholic acid	CDCA
Free deoxycholic acid	DCA
Free lithocholic acid	LCA
Free ursodeoxycholic acid	UDCA
Free hyocholic acid	HCA
Free hyodeoxycholic acid	HDCA
Free alpha-muricholic acid	αMCA
Free beta-muricholic acid	βMCA
Free omega-muricholic acid	ωMCA
Glyco-cholic acid	GCA
Glyco-chenodeoxycholic acid	GCDCA
Glyco-deoxycholic acid	GDCA
Glyco-lithocholic acid	GLCA
Glyco-ursodeoxycholic acid	GUDCA
Glyco-hyocholic acid	GHCA
Glyco-hyodeoxycholic acid	GHDCA
Tauro-cholic acid	TCA
Tauro-chenodeoxycholic acid	TCDCA
Tauro-deoxycholic acid	TDCA
Tauro-lithocholic acid	TLCA
Tauro-ursodeoxycholic acid	TUDCA
Tauro-hyocholic acid	THCA
Tauro-hyodeoxycholic acid	THDCA
Tauro-alpha-muricholic acid	TαMCA
Tauro-beta-muricholic acid	TβMCA
Tauro-omega-muricholic acid	TωMCA
Total CDCA	CDCA + GCDCA + TCDCA
Total DCA	DCA + GDCA + THCA
Total LCA	LCA + GLCA + TLCA
Total UDCA	UDCA + GUDCA + TUDCA
Total HCA	HCA + GHCA + THCA
Total HDCA	HDCA + GHDCA + THDCA
Total primary BAs	Total CA + Total CDCA + Total αMCA + Total βMCA
Total secondary BAs	Total DCA + Total LCA + Total UDCA + Total HCA + Total HDCA
Total free BAs	CA + CDCA + DCA + LCA + UDCA + HCA + HDCA
Total conjugated	GCA + GCDCA + GDCA + GLCA + GUDCA + GHCA + GHDCA + TCA + TCDCA + TDCA + TLCA + TUDCA + THCA + THDCA
Total primary	CA + CDCA + TCA + TCDCA + GCA + GCDCA + αMCA + βMCA
Total secondary	DCA + LCA + UDCA + HCA + HDCA + ωMCA + TDCA + TLCA + TUDCA + THCA + THDCA + TωMCA
Total BAs	Total Free BAs + Total Conjugated BAs
Total 12αOH BAs	Total CA + Total DCA
Total non12αOH BAs	Total CDCA + Total LCA + Total UDCA + Total HCA + Total HDCA
Ratio 12αOH	Total 12αOH BAs / Total non12αOH BAs

Table S5 : Relative abundance of families and genera evaluated by 16S rRNA MiSeq sequencing in cecal contents of lean (LFD) and obese (HFD) mice treated or not with PI10 (HFD PI10) or the mixture (HFD + LA804/LA806).

	LFD	HFD	HFD + PI10	HFD + LA804/ LA806
Actinobacteria	402.25 ± 173.78	79 ± 24.97	331.62 ± 99.19	792.75 ± 261.53
Alcaligenaceae	108.75 ± 70.02	41.875 ± 25.72	13 ± 10.62	37.375 ± 11.13
Bifidobacteriaceae	306.25 ± 136.91	21 ± 7.49	225.5 ± 72.92	203.625 ± 25.41
Clostridiaceae	48.75 ± 18.74	94.625 ± 31.99	269.125 ± 78.29	13.5 ± 7.28
Coriobacteriaceae	96 ± 42.15	58 ± 14.74	106.125 ± 28.61	589.125 ± 250.92
Peptostreptococcaceae	3 ± 1.68	36.125 ± 16.16	23.125 ± 10.52	4.25 ± 2.20
Ruminococcaceae	1482.25 ± 619.68	1107.25 ± 78.97	1986.25 ± 252.09	1414.375 ± 133.40

	LFD	HFD
Bifidobacteriaceae	306.25 ± 136.91	21 ± 7.49
Peptostreptococcaceae	3 ± 1.68	36.125 ± 16.16
Streptococcaceae	272.25 ± 76.41	707 ± 77.33
Eubacterium rectale	12.25 ± 3.01	0.5 ± 0.37
Bifidobacterium	306.25 ± 273.82	21 ± 7.49
Blautia	78 ± 91.72	557.625 ± 125.80
Eisenbergiella	31.5 ± 9.32	1.125 ± 0.47
Faecalibacterium	535.75 ± 999.23	0.25 ± 0.25
Lachnospiraceae NK4A136	944 ± 503.80	404.375 ± 86.95
Lactococcus	269.25 ± 149.97	700.75 ± 77.76
Oscillibacter	38.25 ± 32.11	129.625 ± 13.49
Parabacteroides	16 ± 5.35	141.75 ± 45.43
Peptoclostridium	3 ± 3.36	36.125 ± 16.16
Peptococcus	4.75 ± 4.92	14.625 ± 2.54
Rikenellaceae RC9 gut	397 ± 350.68	74.875 ± 16.96
Ruminiclostridium	17.75 ± 13.04	84.875 ± 20.24
Ruminiclostridium 9	139.75 ± 64.2	359 ± 43.09
Ruminococcaceae UCG-014	302.5 ± 129.97	49.125 ± 16.39
Ruminococcus 1	0.25 ± 0.5	21.125 ± 7.18
Turicibacter	0 ± 0.00	29.625 ± 9.46

	HFD	HFD-PI10
Actinobacteria	79 ± 24.97	331.625 ± 99.19
Bifidobacteriaceae	21 ± 7.49	225.5 ± 72.92
Ruminococcaceae	1107.25 ± 78.97	1986.25 ± 252.09
Bifidobacterium	21 ± 7.49	225.5 ± 72.92
Blautia	557.625 ± 125.8	207.75 ± 40.48
Enterorhabdus	33.125 ± 8.48	73.625 ± 16.73
Lachnoclostridium	446.125 ± 112.71	167.625 ± 40.25
Parabacteroides	141.75 ± 45.43	36 ± 15.10
Roseburia	220.125 ± 39.73	36.125 ± 6.82
Ruminococcaceae UCG-014	49.125 ±16.39	560.625 ± 143.67
Ruminococcus 1	21.125 ± 7.18	143.25 ± 42.09

	HFD	HFD + LA804/ LA806
Actinobacteria	79 ± 24.97	792.75 ± 261.53
Bifidobacteriaceae	21 ± 26.16	203.625 ± 25.41
Clostridiaceae 1	94.625 ± 25.87	13.5 ± 7.28
Coriobacteriaceae	58 ± 16.87	589.125 ± 250.49
Bifidobacterium	21 ± 7.49	203.625 ± 25.41
Clostridium sensu stricto 1	94.625 ± 90.48	13.5 ± 7.28
Coriobacteriaceae UCG-002	22.875 ± 28.06	460.125 ± 244.42
Oscillibacter	129.625 ± 38.15	59.875 ± 7.78

Data are expressed as mean ± SEM values.

Table S6. **Portal plasma BA concentrations.** Means (and SEM), p-values from non-parametric Mann-Whitney test. p-values in bold are statistically significant.

nM	LFD	HFD	HFD-P10	HFD-LA804/LA806	p (HFD vs LFD)	p (HFD-P10 vs HFD)	p (HFD-LA804/LA806-vs HFD)
**CA**	2921 (1664)	1521 (1383)	1547 (1087)	1512 (1460-	0.23	0.80	>0.99
**CDCA**	297 (198)	47 (74)	61 (104)	38 (56)	**0.036**	0.76	0.75
**DCA**	407 (244)	549 (360)	591 (261)	471 (298)	0.65	0.69	0.46
**LCA**	0 (0)	0 (0)	0 (0)	0 (0)	>0.99	>0.99	>0.99
**UDCA**	856 (318)	286 (233)	101 (118)	372 (342)	**0.009**	0.28	0.61
**αMCA**	656 (499)	240 (468)	288 (376)	673 (1164)	**0.036**	0.39	0.31
**βMCA**	4848 (994)	1406 (1989)	1131 (1390)	1945 (2327)	**0.042**	0.78	0.54
**ωMCA**	2103 (1071)	723 (903)	583 (571)	1099 (1659)	0.11	0.96	0.46
**HCA**	0 (0)	0 (0)	0 (0)	0 (0)	>0.99	>0.99	>0.99
**HDCA**	99 (127)	63 (89)	124 (115)	58 (94)	0.5	0.14	0.84
**TCA**	2045 (1121)	863 (670)	741 (584)	1006 (813)	0.11	0.87	0.87
**TCDCA**	141 (93)	102 (97)	126 (100)	87 (66)	0.53	0.69	>0.99
**TDCA**	119 (63)	256 (148)	245 (124)	258 (189)	0.16	>0.99	0.96
**TLCA**	0 (0)	0 (0)	0 (0)	0 (0)	>0.99	>0.99	>0.99
**TUDCA**	51 (19)	50 (40)	48 (24)	53 (49)	0.93	>0.99	0.96
**TαMCA**	937 (516)	495 (503)	587 (483)	670 (588)	0.11	0.64	0.40
**TβMCA**	1206 (338)	618 (811)	500 (416)	759 (595)	0.22	0.87	0.51
**TωMCA**	110 (114)	69 (123)	215 (268)	224 (268)	0.5	0.37	0.11
**THCA**	0 (0)	0 (0)	0 (0)	0 (0)	>0.99	>0.99	>0.99
**THDCA**	7 (14)	17 (14)	41 (51)	17 (27)	0.17	0.41	0.33

Table S7. BA proportions in cecal contents. Means (and SEM). p-values from non-parametric Mann-Whitney test. P-values in bold are statistically significant.

%	LFD	HFD	HFD-P10	HFD-LA804/LA806	p (HFD vs LFD)	p (HFD-P10 vs HFD)	p (HFD-LA804/LA806-vs HFD)
**CA**	5 (2)	6 (2)	6 (2)	6 (4)	0.46	0.80	0.83
**CDCA**	0 (0)	0 (0)	0 (0)	0 (0)	>0.99	>0.99	>0.99
**DCA**	17 (2)	32 (6)	29 (6)	28 (10)	**0.006**	0.24	0.33
**LCA**	0 (0)	1 (0)	0 (0)	0 (0)	0.24	0.13	0.10
**UDCA**	4 (3)	2 (2)	3 (1)	4 (2)	0.40	0.47	0.15
**αMCA**	10 (3)	9 (4)	12 (4)	13 (4)	0.55	0.16	0.10
**βMCA**	33 (7)	16 (6)	21 (7)	26 (7)	**0.012**	0.16	**0.020**
**ωMCA**	24 (3)	25 (4)	18 (2)	16 (3)	0.69	**0.0009**	**0.002**
**HCA**	0 (0)	0 (0)	0 (0)	0 (0)	>0.99	>0.99	>0.99
**HDCA**	4 (1)	5 (2)	6 (2)	4 (1)	0.31	0.50	0.09
**TCA**	0 (0)	1 (0)	0 (0)	0 (0)	>0.99	>0.99	0.56
**TCDCA**	0 (0)	0 (0)	0 (0)	0 (0)	>0.99	>0.99	>0.99
**TDCA**	0 (0)	0 (0)	0 (0)	0 (0)	>0.99	>0.99	>0.99
**TLCA**	0 (0)	0 (0)	0 (0)	0 (0)	>0.99	>0.99	>0.99
**TUDCA**	0 (0)	0 (0)	0 (0)	0 (0)	>0.99	>0.99	>0.99
**TαMCA**	1 (0)	0 (0)	1 (0)	0 (0)	0.55	>0.99	>0.99
**TβMCA**	1 (0)	1 (1)	2 (1)	1 (0)	0.83	0.11	>0.99
**TωMCA**	1 (0)	1 (0)	1 (1)	0 (0)	>0.99	0.48	0.27
**THCA**	0 (0)	0 (0)	0 (0)	0 (0)	>0.99	>0.99	>0.99
**THDCA**	0 (0)	0 (0)	0 (0)	0 (0)	>0.99	>0.99	>0.99

Supplementary Figures

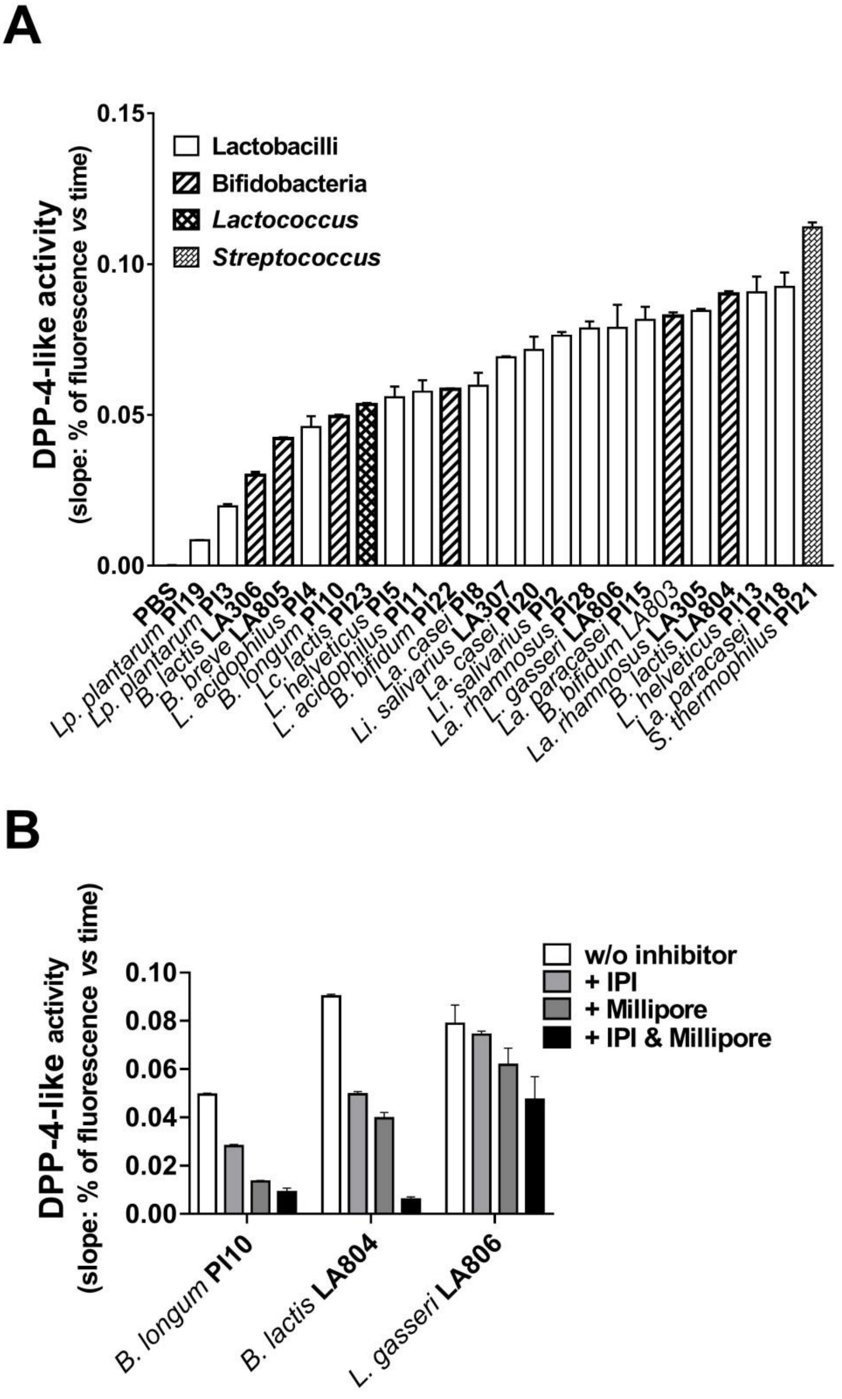

Figure S1 : DPP-4 like activity of the strains. (**A**) DPP-4-like activity of the strains evaluated by their ability to cleave the Gly-Pro-AMC which led to fluorescence emission. (**B**) DPP-IV-like activity of the three strains used *in vivo* in presence or absence of DPP-IV inhibitors: IPI and Millipore. The DPP-IV-like activity is expressed by the slope from the linear regression of the percentage of fluorescence as a function of time (n=3).
